# Hematological inflammatory biomarkers in patients with alcohol and cocaine use disorders

**DOI:** 10.47626/2237-6089-2023-0723

**Published:** 2025-09-18

**Authors:** Andressa Goldman Ruwel, Juliana Nichterwitz Scherer, Daiane Silvello, Felix Henrique Paim Kessler, Lisia von Diemen, Jaqueline Bohrer Schuch

**Affiliations:** 1 Centro de Pesquisa em Álcool e Drogas Hospital de Clínicas de Porto Alegre Universidade Federal do Rio Grande do Sul Porto Alegre RS Brazil Centro de Pesquisa em Álcool e Drogas, Hospital de Clínicas de Porto Alegre, Universidade Federal do Rio Grande do Sul (UFRGS), Porto Alegre, RS, Brazil.; 2 Programa de Pós-Graduação em Saúde Coletiva Universidade do Vale do Rio dos Sinos São Leopoldo RS Brazil Programa de Pós-Graduação em Saúde Coletiva, Universidade do Vale do Rio dos Sinos, São Leopoldo, RS, Brazil.; 3 Programa de Pós-Graduação em Psiquiatria e Ciências do Comportamento Departamento de Psiquiatria UFRGS Porto Alegre RS Brazil Programa de Pós-Graduação em Psiquiatria e Ciências do Comportamento, Departamento de Psiquiatria, UFRGS, Porto Alegre, RS, Brazil.

**Keywords:** Neutrophil-lymphocyte ratio, alcohol use disorder, cocaine use disorder, inflammatory

## Abstract

**Objective:**

Neutrophil-lymphocyte ratio (NLR), monocyte-to-lymphocyte ratio (MLR), and platelets-lymphocyte ratio (PLR) are biomarkers easy-to-obtain and could be used in clinical practice to verify an inflammatory status and are associated with alcohol use disorder (AUD) and cocaine use disorder (CUD). Our aim was to compare NLR, MLR, and PLR among men with AUD and CUD and to assess the relationship between these biomarkers and addiction-related outcomes.

**Methods:**

This is a cross-sectional study comprising 979 inpatient men diagnosed with substance use disorder (391 with AUD and 588 with CUD) under hospital treatment for drug addiction.

**Results:**

Individuals with AUD had higher NLR and MLR (p = 0.041, p < 0.001 respectively) compared to individuals with CUD. In the AUD group, positive correlations between age and MLR (r = 0.111; p = 0.029), NLR and liver enzymes alanine transaminase (ALT) and aspartate transaminase (AST) (r = 0.103, p = 0.043; r = 0.155, p = 0.002; respectively), and MLR and ALT, AST and gamma-glutamyl transferase (GGT) levels were observed (r = 0.173, p = 0.001; r = 0.242, p < 0.001; r = 0.167, p = 0.001, respectively). Individuals with CUD showed a positive correlation between age and NLR (r = 0.113; p = 0.006). The presence of clinical comorbidities, human immunodeficiency virus (HIV), hepatitis C virus (HCV) and syphilis were not associated with NLR, MLR, and PLR (p > 0.05).

**Conclusion:**

These biomarkers are a rapid and inexpensive way to assess the effects of substance use on the inflammatory profile. Our findings contribute with valuable insights into the distinctive inflammatory profiles associated with AUD and CUD. These insights could guide further research and the development of more studies, which could include control groups, in order to refine the clinical applicability of these biomarkers.

## Introduction

Alcohol consumption is associated with several negative outcomes, including social, legal and health aspects, increases the susceptibility to respiratory syndromes, liver diseases and sepsis,^1^ and it is a risk factor for comorbid illnesses, and premature mortality.^2^ The prevalence of alcohol use disorder (AUD) is about 4% in the Americas, being also a relevant problem worldwide.^3^ Cocaine use disorder (CUD) is also a condition that leads to health complications, with high rates of clinical and mental health morbidity and mortality. In fact, mortality among cocaine users occurs four to eight times more often than in the general population.^4^ Individuals who use cocaine and crack often present myocardial infarction, stroke, overdose, violent behavior, and legal problems.^5,6^ Brazil is one of the countries with the highest prevalence of lifetime cocaine and crack use, estimated at 3.1 and 0.9%, respectively.^7^

Alcohol and cocaine use are involved with changes in the regulation and signaling of the immune system, through immunodeficiency and autoimmunity mechanisms,^1,8^ and associated with inflammatory and neurodegenerative processes.^9^ Alcohol misuse can suppress blood cell production, increases the risk of infections,^10^ and cytokines levels, such as interleukin (IL)-6.^11^ Moreover, ethanol intoxication inhibits the release of neutrophils in inflammatory processes.^12^ Similarly, cocaine abuse may also lead to increased production of cytokines and changes in lymphocyte subsets.^13^ Cocaine administration increases the number of CD4+, CD8+, T helper (Th)1, Th2, Th17 and lymphocytes changes,^14,15^ and long-term abuse can contribute to neurotoxicity.^16^ The smoked form of cocaine is crack, which is characterized by its rapid effects on the central nervous system, presenting distinct patterns of potential addiction and health implications compared to snorting cocaine.^17^ This scenario indicates that substance abuse can lead to neuroinflammatory states that could deteriorate the nervous system functioning, and lead to negative implications in clinical and treatment outcomes.

Neutrophils, monocytes, and platelets have essential roles in the systemic inflammatory response and through their count and relationship with lymphocytes have been used as markers of inflammation. Neutrophils are the most abundant type of leukocytes and are related to acute and chronic inflammation and infectious processes.^18^ Monocytes are responsible for the control and clearance of infectious diseases, differentiating into macrophage and dendritic cells.^19^ Similarly, platelets play a major role in blood clotting and inflammation by detecting and adhering to the injured endothelium, helping to resolve the local damage.^20^ Studies have investigated these cells through their ratio with lymphocytes, and the markers are denominated neutrophil-lymphocyte ratio (NLR), monocyte-to-lymphocyte ratio (MLR), and platelets-lymphocyte ratio (PLR). These specific biomarkers are easy-to-obtain, reproducible, have low-cost and have been widely investigated as inflammatory parameters for several diseases.

NLR was the first of these biomarkers used to assess the intensity of stress and inflammation.^21^ Evidence showed an association of higher NLR, PLR, and MLR with psychiatric disorders and behaviors, including suicide attempts,^22^ major depressive disorder,^23^ bipolar disorder, and schizophrenia.^24^ Concerning addictive behaviors, higher NLR was observed in older individuals with CUD,^14^ in individuals with AUD^25^ and with heroin dependence.^26^ In addition, a higher MLR was observed in individuals with AUD^25^ while higher PLR was detected in individuals with heroin dependence.^26^ Although PLR was not directly associated with AUD, a negative correlation was observed with years of alcohol use.^27^ On the other hand, lower PLR, MLR, and percentage of monocytes were observed in individuals with opioid use disorders compared to the healthy controls.^28^

Overall, studies conducted so far have demonstrated changes in these inflammatory parameters in individuals with substance use disorder (SUD) when compared to controls, with findings that vary according to the type and severity of substance use. In this sense, it is essential to assess and compare NLR, MLR, and PLR biomarkers among different substances, especially in crack users. Therefore, the aims of the present study were: 1) to compare NLR, MLR, and PLR in inpatient men with AUD and CUD (specifically smoked cocaine – crack use); 2) to assess the relationship between these biomarkers and addiction-related outcomes; 3) to assess the influence of clinical characteristics on NLR, MLR, and PLR in individuals with SUD.

## Material and Methods

### Sample and procedures

This is a cross-sectional study, which included men diagnosed with SUD recruited at a male inpatient unit specialized in the treatment of drug addiction in the Hospital de Clínicas de Porto Alegre, Southern Brazil. All individuals were under similar detoxification treatment, between 2012 and 2020. Inclusion criteria were aged between 18 and 65 years, having a diagnosis of AUD or CUD (cocaine smoking/crack) according to the Diagnostic and Statistical Manual of Mental Disorders, 5th version (DSM-5) criteria,^29^ and authorized access to medical record data. Participants who had severe cognitive deficits that could impair the patient’s capacity to respond to the instruments were excluded (Mini Mental State Examination [MMSE] < 13). The total sample consisted of 1,096 individuals, but after subsequent exclusions based on the selection criteria described above, 979 inpatients (391 with AUD and 588 with CUD) were included in the analyses. Individuals answered the research protocol during the 1st days of hospitalization. Undergraduate students received training for applying this protocol and were supervised by a senior researcher (psychologist or psychiatrist). The research protocol included a sociodemographic questionnaire, which provides data regarding age, skin color, schooling, marital status, body mass index (BMI), chronic diseases, family history of substance use, suicide attempt, and history of substance use. The Structured Clinical Interview for DSM-IV Axis I (SCID-I) was applied to assess psychiatric disorders following DSM guidelines^29^ as well as the Addiction Severity Index 6th version (ASI-6),^30^ which is a semi-structured interview designed to collect information that could provide important insights about the substance abuse, medical and occupational status, legal, socio-family and psychiatric aspects. The scores generated from ASI-6 indicate severity of problems in these different areas. Clinical comorbidities were assessed by self-reports and medical records. The use of medications was extracted from hospital records.

### Ethical considerations

This study was approved by the Research Ethics Committee of the Hospital de Clínicas de Porto Alegre (HCPA) (no. 2014-0249) and was carried out in accordance with the Declaration of Helsinki. All participants signed informed consent before inclusion in the study.

### Blood collection and analyses

The peripheral blood sample was collected in a tube with ethylenediaminetetraacetic acid (EDTA) anticoagulant on the 1st day of hospitalization, during the morning (7 a.m.-8 a.m.). All participants were fasting for 8 hours before blood collection. Leucocytes were analyzed by flow cytometry, using the Sysmex XN-series analyzer in the hematology laboratory at the HCPA, following guidelines and standard protocols. The results of neutrophils, lymphocytes, monocytes, and platelets were collected from hospital records. Participants who had leukocytosis (> 11,000 cells) or leukopenia (< 4,000 cells) were excluded (n = 117), resulting in 979 individuals included in the analyses. NLR, MLR, and PLR were calculated using the complete blood test and dividing the value of the neutrophil (or monocyte or platelet) count by the number of lymphocytes.

Aspartate transaminase (AST), alanine transaminase (ALT), and gamma-glutamyl transferase (GGT) are enzymes that may indicate the severity of liver function and damage and were also measured by standard laboratory protocols at the HCPA. In addition, the presence of antibodies to human immunodeficiency virus (HIV), hepatitis B, and syphilis were analyzed following HCPA standard protocols using microparticle chemiluminescent immunoassays.

### Statistical analyses

Statistical analyses were performed in SPSS version 18. The significance level considered was 0.05. Continuous variables were assessed for normality using a histogram and the Kolmogorov-Smirnov test. All continuous variables presented an asymmetrical distribution and were expressed by the median and interquartile range (IQR). Categorical data was presented by absolute and relative frequency. Sociodemographic and clinical characteristics were compared between AUD and CUD using chi-square and Mann-Whitney *U* tests.

The comparison of NLR, MLR, and PLR between individuals with AUD and CUD was performed using the Mann-Whitney *U* test. Linear regression models were performed to evaluate the association between these biomarkers and the groups (AUD and CUD), also adjusting these associations for potential confounders (age, skin color, use of anti-inflammatory medications, presence of HIV, syphilis, hepatitis C virus [HCV], and chronic diseases, including cardiovascular, diabetes, history of stroke, cirrhosis, autoimmune, and kidney and respiratory diseases). The relationship between NLR, MLR, and PLR and addiction-related outcomes (including years of substance use, ASI scores and hospitalization days), as well as with clinical variables (i.e., age, liver enzymes, presence of chronic and infectious diseases) were analyzed through Spearman correlation or Mann Whitney *U* test. The assessment of the relationship between hospitalization days and inflammatory biomarkers relies on the possibility that individuals with more severe inflammatory profiles might require extended hospitalization (more hospitalization days), reflecting a more complex clinical condition.

## Results

Individuals with AUD were older, predominantly married, with lower education level, and remained hospitalized for longer periods than individuals with CUD. These individuals also presented a higher prevalence of use of anti-inflammatory medications (Table 1).

**Table 1:** Comparison of sociodemographic characteristics between individuals with AUD and CUD

	AUD (n = 443)	CUD (n = 653)	Statistics	p-value
Age	51.0 (45.0-57.0)	35.0 (28.0-44.0)	-17.941	< 0.001
Hospitalization (days)	31.0 (16.0-43.0)	10.0 (5.0-26.0)	-12.932	< 0.001
Marital status (married)	110 (28.5)	99 (16.9)	17.998	< 0.001
Education (high school or bachelor’s degree)	137 (35.8)	248 (42.6)	4.228	0.040
Skin color (white)	243 (62.3)	378 (64.5)	0.398	0.528
HIV, HCV, or syphilis (yes)	46 (11.8)	97 (16.5)	3.845	0.050
Chronic diseases* (yes)	115 (29.9)	144 (27.1)	0.703	0.402
Anti-inflammatory medications^†^ (yes)	56 (14.3)	31 (5.3)	22.651	< 0.001

AUD = alcohol use disorder; CUD = cocaine use disorder; HCV = hepatitis C virus; HIV = human immunodeficiency virus.

Data is presented as median (interquartile range [IQR]) or n (%).

* Chronic diseases: cardiovascular diseases, diabetes, stroke, cirrhosis, renal disease, respiratory problems, and autoimmune diseases.

^†^ Anti-inflammatory medications: acetylsalicylic acid dipyrone, prednisone, ibuprofen, promethazine.

**Figure 1:**
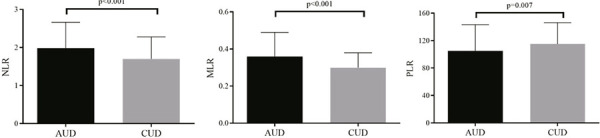
Comparison of hematological inflammatory biomarkers between alcohol use disorder (AUD) and cocaine use disorder (CUD) groups. Data presented as median and interquartile range (IQR). Neutrophil-lymphocyte ratio (NLR)-AUD = 1.98 (1.44-2.66), CUD = 1.70 (1.27-2.28); monocyte-lymphocyte ratio (MLR)-AUD = 0.36 (0.25-0.49), CUD = 0.29 (0.23-0.38); platelet-lymphocyte ratio (PLR)-AUD = 105.52 (79.74-143.88), CUD = 115.22 (92.34-146.20).

The analyses comparing inflammatory biomarkers and addiction groups demonstrated that individuals with AUD have high levels of NLR (z = -4.923; p < 0.001) and MLR (z = -6.355; p < 0.001), and lower PLR (z = 2.718; p = 0.007) compared to participants with CUD (Figure 1). Then, linear regression models were performed, and the factor group (AUD *versus* CUD) was associated with NLR and MLR biomarkers even adjusting for confounding variables (p = 0.041, p < 0.001 respectively) (Table 2).

**Table 2:** Linear regression analysis adjusting for potential confounders

	Beta	95%CI	t-statistics	p-value
NLR				
AUD (reference)				
CUD	-0.081	-0.152, -0.003	-2.050	0.041
				
MLR				
AUD (reference)				
CUD	-0.187	- 0.231, -0.095	-4.734	< 0.001
				
PLR				
AUD (reference)				
CUD	0.040	- 0.032, 0.096	0.992	0.321

95%CI = 95% confidence interval; AUD = alcohol use disorder; CUD = cocaine use disorder; MLR = monocyte-lymphocyte ratio; NLR = neutrophil-lymphocyte ratio; PLR = platelet-lymphocyte ratio.

Linear regression analyses were adjusted for age, skin color, presence of clinical comorbidities (cardiovascular diseases, diabetes, stroke, cirrhosis, renal disease, respiratory problems, and autoimmune diseases), human immunodeficiency virus (HIV), hepatitis C virus (HCV) and syphilis, and use of anti-inflammatory medications. Adjusted R-squared for each model: NLR = 0.046, MLR = 0.049, PLR = 0.008.

Individuals with CUD showed a positive but weak correlation between age and NLR (r = 0.113; p = 0.006) (Table 3). No association was observed between NLR, MLR, and PLR and addiction severity (ASI-6 score, p > 0.05) (Table 3), years of cocaine/crack use (p > 0.05) (Table 3), and hospitalization days (p > 0.05) (Table 3). Individuals with AUD showed a positive correlation between age and MLR (r = 0.111; p = 0.029) (Table 3). NLR, MLR, and PLR were not correlated to addiction severity (p > 0.05) (Table 3), years of alcohol use (p > 0.05) (Table 3), and hospitalization days (p > 0.05) (Table 3). Furthermore, no difference was observed regarding NLR, MLR, and PLR biomarkers when comparing individuals with and without clinical comorbidities, use of anti-inflammatory drugs, and comparing the presence and absence of HIV, HCV and/or syphilis in both groups – AUD and CUD (p > 0.05) (Supplementary Tables S1 and S2).

**Table 3:** Spearman correlation analyses between NLR, MLR, PLR and addiction-related outcomes, age and liver enzymes in individuals with AUD and CUD

	AUD (n = 443)			CUD (n = 653)		
	NLR	MLR	PLR	NLR	MLR	PLR
Age (years)	r = 0.083 p = 0.100	**r = 0.111 p = 0.029**	r = -0.040 p = 0.436	**r = 0.113 p = 0.006**	r = 0.003 p = 0.938	r = -0.080 p = 0.053
Hospitalization (days)	r = 0.002 p = 0.996	r = 0.075 p = 0.140	r = 0.015 p = 0.763	r = 0.019 p = 0.644	r = 0.026 p = 0.528	r = -0.021 p = 0.610
Years of substance use	r = 0.007 p = 0.913	r = 0.016 p = 0.796	r = 0.030 p = 0.645	r = 0.046 p = 0.386	r = -0.037 p = 0.490	r = 0.001 p = 0.982
ASI scores	r = -0.009 p = 0.890	r = -0.035 p = 0.566	r = -0.085 p = 0.169	r = -0.087 p = 0.086	r = -0.046 p = 0.364	r = -0.071 p = 0.159
AST levels	**r = 0.155 p = 0.002**	**r = 0.242 p < 0.001**	r = -0.030 p = 0.556	-	-	-
ALT levels	**r = 0.103 p = 0.043**	**r = 0.173 p = 0.001**	r = -0.036 p = 0.490	-	-	-
GGT levels	r = 0.067 p = 0.199	**r = 0.167 p = 0.001**	r = -0.061 p = 0.245	-	-	-

ALT = alanine transaminase; ASI = Addiction Severity Index; AST = aspartate aminotransferase; AUD = alcohol use disorder; CUD = cocaine use disorder; GGT = gamma-glutamyl transferase; MLR = monocyte-lymphocyte ratio; NLR = neutrophil-lymphocyte ratio; PLR = platelet-lymphocyte ratio.

Bold type indicates significant associations (p < 0.05).

Additional analyses were performed in participants with AUD considering the concentration of liver enzymes. A positive correlation between NLR and ALT and AST levels was observed (r = 0.103, p = 0.043; r = 0.155, p = 0.002; respectively) (Table 3). Similarly, MLR was positively correlated to ALT, AST, and GGT levels (r = 0.173, p = 0.001; r = 0.242, p < 0.001; r = 0.167, p = 0.001, respectively) (Table 3). On the other hand, PLR was not correlated to ALT, AST, GGT (p > 0.05) (Table 3).

## Discussion

This is the first study that compared NLR, MLR, and PLR in individuals with AUD and CUD and explored possible influences on addiction-related outcomes, as far as we know. In our study, individuals with AUD presented predominantly higher concentrations of NLR and MLR, which suggests a more pronounced inflammatory state. It is important to highlight that our study also corroborated that advanced age is a factor that affects inflammation, as detected by our correlation analyses. In addition, higher levels of NLR and MLR were positively correlated to liver enzymes, such as ALT and AST. The sample analyzed in this study consists of individuals with addiction in the initial period of hospital admission and early abstinence. This characteristic must be taken into account when interpreting the results found.

Few studies have assessed these biomarkers in AUD, only comparing them to healthy controls, and showing mixed findings.^25,27^ The pattern and amount of alcohol use may vary between studies, which may reflect the mixed results found. It can be suggested that individuals who make sporadic or acute use of alcohol may not have substantial effects on peripheral inflammatory markers, while chronic and frequent use has more clinical and biological impacts. In our study, individuals with AUD had chronic use, as evidenced by the high mean years of frequent alcohol use. On the other hand, this pattern of chronic use prevented us from detecting possible nuances of the effects of alcohol on these biomarkers. Moreover, these individuals presented higher levels of NLR and MLR compared to individuals with CUD, who had fewer years of substance use. The association between group (AUD *versus* CUD) and NLR and MLR was significant even when adjusting the model or potential confounders, such as age, and clinical comorbidities. This demonstrates that alcohol use itself may have affected inflammatory processes in a more pronounced way than crack use.

Several pieces of evidence have shown that alcohol interferes with the immune system and inflammation through different pathways. Alcohol use has been associated with changes in inflammatory biomarkers, particularly cytokines. A recent meta-analysis showed that only IL-6 levels, but no other cytokines, were significantly higher in individuals with AUD compared to controls.^11^ In addition, studies indicated that alcohol activates a hyper-ramified microglia form, which has been related to cytokine release.^31^ In rats exposed to ethanol, an increased activation and proliferation of microglia was also observed.^32^ Furthermore, in the liver, Kupffer cells and hepatocytes are stimulated by alcohol use to generate free radicals and cytokines.^33^ Therefore, oxidative damage also contributes to this scenario, since it activates the immune response, exacerbating the inflammatory state.^34,35^ Alcohol also favors the translocation of bacteria from the intestinal lumen to periphery, and these leaked bacterial products can cause inflammation in the liver and release pro-inflammatory cytokines into systemic circulation.^36^ Animal studies also corroborate the relationship between alcohol and inflammation. For instance, higher levels of IL-6 were associated with increased preference or ingestion of alcohol.^36,37^

Individuals with SUD commonly present other clinical and psychiatric comorbidities. These pathologies have also been associated with alterations in inflammatory biomarkers.^22,23^ Despite the high clinical complexity associated with alcohol use, additional analyzes demonstrated that the presence of chronic diseases does not directly interfere or contribute with the levels of these inflammatory biomarkers in this population. These findings reinforce that chronic alcohol use per se has a substantial impact on inflammatory processes. On the other hand, this inflammatory state associated with substance use can contribute to or worsen the clinical presentation of other chronic diseases, such as cardiovascular, renal, and psychiatric diseases, increasing susceptibility to chronic morbidity, disability, and frailty.^38^ In this sense, the evaluation of NLR, MLR, and PLR and their relationship with addiction may help to provide a risk severity evaluation, intensity of treatment, and a brighter clinical prognosis. The association of inflammatory markers with adverse health outcomes indicates that targeting treatment to reduce inflammation can mitigate the severity of other diseases.^39^

Age is another factor associated with inflammation processes, which is in line with our findings. Studies indicated that higher levels of basal immune activity occur during aging. For instance, other inflammatory markers, such as IL-6, tumor necrosis factor alpha and C-reactive protein, were previously related to age-related chronic diseases.^39^ The inflammaging state is the result of the chronic physiological stimulation of the innate immune system in older people, where an excessive amount of proinflammatory cytokines is secreted.^40^ Moreover, advanced age associated with alcohol consumption has immunomodulatory effects by changing neutrophil recruitment and its ability to enter tissues.^41,42^ The use of substances can also weaken neutrophils and its ability to respond to infections.^43^ Ultimately, the metabolism of alcohol is slower in older adults, leading to more prolonged and worsened effects.^44^

In our study, we also explored other clinical and peripheral variables in participants with AUD. Positive correlations were detected between NLR, MLR, and liver enzymes, including AST and ALT. The chronic use of alcohol leads to hepatocellular injury and inflammation. In this sense, changes in liver function markers, such as AST and ALT, have been related to advanced alcoholic liver disease.^45^ In addition, alcohol use increases the circulation of lipopolysaccharide (LPS), which activates Kupffer cells in the liver and stimulates the innate immune system to produce cytokines and inflammation.^46^ Although most of the studies indicate an increase in cytokines during this process, we suggest that changes in other inflammatory biomarkers, particularly NLR and MLR, can also indicate liver damage and greater severity of alcohol abuse.

Our results corroborate other findings that suggest that inflammation plays a significant role in addiction, particularly in AUD, and may provide insight into the development of novel treatment modalities focused on preventing or mitigating inflammation as an integral component of addiction treatment. Considering the limited efficacy of anti-inflammatory agents in this context, other therapeutic interventions, such as immunomodulatory therapies, mind-body interventions, or nutritional interventions designed to modulate inflammatory processes directly, may offer promising avenues for enhancing addiction treatment outcomes and the overall well-being of affected individuals. Nonetheless, additional research is warranted to elucidate the underlying mechanisms comprehensively and to advance in the development of personalized and precision therapies.

The study has some limitations that should be considered. The primary limitation of this study is related to the absence of a control group for comparative analysis. However, this fact does not exclude the hypothesis suggesting that substance use may influence alterations in the immune system response. The question of how much these biomarkers are altered in individuals with SUD compared to those without SUD remains an important aspect that warrants further investigation. Also, its cross-sectional design prevents the establishment of causal relationships between biomarkers and addiction-related outcomes, warranting the need for longitudinal studies. The generalizability of the findings is limited since the study was conducted in a specific male inpatient unit focused on drug addiction treatment in Southern Brazil, and results may differ or vary when analyzing female inpatients or other individuals with a different pattern of substance use. The study also focused on a limited number of inflammatory markers, excluding other potential markers that could provide a more comprehensive understanding of the inflammatory response.Also, addiction is a complex behavior, and substance abusers often present clinical and psychiatric comorbidities or even are involved in stressful situations, which could also interfere in inflammatory parameters. The exclusion of individuals with pathologies other than SUD could lead to a bias in the selection of samples that are not representative of this vulnerable population. Moreover, it is important to mention that the diagnosis of comorbid psychiatric disorders in SUD s can be unreliable during a short inpatient treatment, as observed in our sample. Psychiatric symptoms can be drug-induced and mimic another psychiatric diagnosis, and therefore, were not explored in our analyses.

## Conclusion

In conclusion, we observed that individuals with AUD presented higher levels of inflammatory biomarkers (NLR and MLR) when compared to individuals with CUD. This is consistent with studies indicating elevated levels of specific inflammatory biomarkers, including NLR, MLR, and PLR, in psychiatric conditions,^21-23^ and substance use.^14,24,25^ However, in contrast to other studies, in the present study we compared the effect of the use of the two main substances used in Brazil (alcohol and crack) on three hematological inflammatory biomarkers (NLR, MLR, and PLR biomarkers), in a sample of male inpatients, in early abstinence with severe addiction. We also evaluated the potential effect of confounding factors, such as medication use, infectious diseases, and clinical comorbidities, strengthening the findings found. The levels of these biomarkers were similar in individuals with or without clinical comorbidities, HIV, HCV, and syphilis, which means the inflammation was probably triggered due to the use of the substance itself. Moreover, the correlations between inflammatory biomarkers (NLR and MLR) and liver enzymes (AST and ALT) in AUD corroborates our hypothesis that substance use alters the immune system response. These biomarkers are a rapid and inexpensive way to assess the effects of substance use on the inflammatory profile, and it may help to detect individuals with a more pronounced inflammatory profile, which may in turn present more complex clinical conditions that require more comprehensive treatment and relapse prevention strategies.

## Supplementary Material

Supplementary Material

## References

[1] Yeligar SM, Chen MM, Kovacs EJ, Sisson JH, Burnham EL, Brown LAS (2016). Alcohol and lung injury and immunity. Alcohol.

[2] Yang P, Tao R, He C, Liu S, Wang Y, Zhang X (2018). The risk factors of the alcohol use disorders-through review of its comorbidities. Front Neurosci.

[3] World Health Organization (2018). Global status report on alcohol and health 2018.

[4] Degenhardt L, Singleton J, Calabria B, McLaren J, Kerr T, Mehta S (2011). Mortality among cocaine users: A systematic review of cohort studies. Drug Alcohol Depend.

[5] Siegel AJ, Sholar MB, Mendelson JH, Lukas SE, Kaufman MJ, Renshaw PF (1999). Cocaine-Induced Erythrocytosis and Increase in von Willebrand Factor: Evidence for Drug-Related Blood Doping and Prothrombotic Effects. Arch Intern Med.

[6] Degenhardt L, Hall W (2012). Extent of illicit drug use and dependence, and their contribution to the global burden of disease. Lancet.

[7] Bastos FIPM (2017). 3rd National survey on drug use by the Brazilian population.

[8] Jacobsen JHW, Hutchinson MR, Mustafa S (2016). Drug addiction: targeting dynamic neuroimmune receptor interactions as a potential therapeutic strategy. Curr Opin Pharmacol.

[9] Periyasamy P, Guo ML, Buch S (2016). Cocaine induces astrocytosis through ER stress-mediated activation of autophagy. Autophagy.

[10] Ballard HS (1997). The Hematological Complications of Alcoholism. Alcohol Health Res World.

[11] Moura HF, Hansen F, Galland F, Silvelo D, Rebelatto FP, Ornell F (2022). Inflammatory cytokines and alcohol use disorder: systematic review and meta-analysis. Brazilian Journal of Psychiatry.

[12] Macgregor RR, Safford M, Shalit M (1988). Effect of Ethanol on Functions Required for the Delivery of Neutrophils to Sites of Inflammation. J Infect Dis.

[13] Coller JK, Hutchinson MR (2012). Implications of central immune signaling caused by drugs of abuse: Mechanisms, mediators and new therapeutic approaches for prediction and treatment of drug dependence. Pharmacol Ther.

[14] Soder HE, Berumen AM, Gomez KE, Green CE, Suchting R, Wardle MC (2020). Elevated neutrophil to lymphocyte ratio in older adults with cocaine use disorder as a marker of chronic inflammation. Clin Psychopharmacol Neurosci.

[15] Zaparte A, Schuch JB, Viola TW, Baptista TAS, Beidacki AS, do Prado CH (2019). Cocaine Use Disorder Is Associated With Changes in Th1/Th2/Th17 Cytokines and Lymphocytes Subsets. Front Immunol.

[16] Büttner A (2011). Review: The neuropathology of drug abuse. Neuropathol Appl Neurobiol.

[17] Vidyasankar G, Souza C, Lai C, Mulpuru S (2015). A severe complication of crack cocaine use. Canadian Respiratory Journal: Journal of the Canadian Thoracic Society.

[18] Rosales C (2018). Neutrophil: A cell with many roles in inflammation or several cell types?. Front Physiol.

[19] Shi C, Pamer EG (2011). Monocyte recruitment during infection and inflammation. Nat Rev Immunol.

[20] Ali RA, Wuescher LM, Worth RG (2015). Platelets: essential components of the immune system. Curr Trends Immunol.

[21] Zahorec R (2001). Ratio of neutrophil to lymphocyte counts-rapid and simple parameter of systemic inflammation and stress in critically ill. CLINICAL REPORT.

[22] Orum MH, Kara MZ, Egilmez OB (2018). Mean platelet volume and neutrophil to lymphocyte ratio as parameters to indicate the severity of suicide attempt. J Immunoassay Immunochem.

[23] Ekinci O, Ekinci A (2017). The connections among suicidal behavior, lipid profile and low-grade inflammation in patients with major depressive disorder: a specific relationship with the neutrophil-to-lymphocyte ratio. Nord J Psychiatry.

[24] Gennaro Mazza M, Clerici M, Rossetti A (2018). A Review of Neutrophil-Lymphocyte, Monocyte-Lymphocyte, and Platelet-Lymphocyte Ratios Use in Psychiatric Disorders. World Journal of Depression and Anxiety.

[25] Kullanim A, Olan B, Tam H, Sayimi K, Ve Lenfositle P, Oranlar I (2019). Complete Blood Count Parameters and Lymphocyte-Related Ratios in Patients with Alcohol Use Disorder. Bagimlilik Dergisi-Journal of Dependence.

[26] Cicek E, Demirel B, Cicek IE, Kirac AS, Eren I (2018). Increased Neutrophil-lymphocyte and Platelet-lymphocyte Ratios in Male Heroin Addicts: A Prospective Controlled Study. Clinical Psychopharmacology and Neuroscience.

[27] Orum MH, Kara MZ (2020). Platelet to lymphocyte ratio (PLR) in alcohol use disorder. J Immunoassay Immunochem.

[28] Orum MH, Kara MZ, Egilmez OB, Kalenderoglu A (2018). Complete blood count alterations due to the opioid use: what about the lymphocyte-related ratios, especially in monocyte to lymphocyte ratio and platelet to lymphocyte ratio?. J Immunoassay Immunochem.

[29] American Psychiatric Association (2022). Diagnostic and Statistical Manual of Mental Disorders.

[30] Kessler F, Cacciola J, Alterman A, Faller S, Souza-Formigoni ML, Cruz MS (2012). Psychometric properties of the sixth version of the Addiction Severity Index (ASI-6) in Brazil. Brazilian Journal of Psychiatry.

[31] Crews FT, Lawrimore CJ, Walter TJ, Coleman LG (2017). The Role of Neuroimmune Signaling in Alcoholism. Neuropharmacology.

[32] McClain JA, Morris SA, Deeny MA, Marshall SA, Hayes DM, Kiser ZM (2011). Adolescent binge alcohol exposure induces long-lasting partial activation of microglia. Brain Behav Immun.

[33] Adachi Y, Bradford BU, Gao W, Bojes HK, Thurman RG (1994). Inactivation of Kupffer cells prevents early alcohol-induced liver injury. Hepatology.

[34] Hill DB, Devalaraja R, Joshi-Barve S, Barve S, McClain CJ (1999). Antioxidants attenuate nuclear factor-kappa B activation and tumor necrosis factor-alpha production in alcoholic hepatitis patient monocytes and rat Kupffer cells, in vitro. Clin Biochem.

[35] Dey A, Cederbaum AI (2006). Alcohol and oxidative liver injury. Hepatology.

[36] Leclercq S, de Timary P, Delzenne NM, Stärkel P (2017). The link between inflammation, bugs, the intestine and the brain in alcohol dependence. Transl Psychiatry.

[37] Leclercq S, Cani PD, Neyrinck AM, Stärkel P, Jamar F, Mikolajczak M (2012). Role of intestinal permeability and inflammation in the biological and behavioral control of alcohol-dependent subjects. Brain Behav Immun.

[38] Ferrucci L, Fabbri E (2018). Inflammageing: chronic inflammation in ageing, cardiovascular disease, and frailty. Nat Rev Cardiol.

[39] Singh T, Newman AB (2011). Inflammatory markers in population studies of aging. Ageing Res Rev.

[40] Franceschi C, Garagnani P, Parini P, Giuliani C, Santoro A (2018). Inflammaging: a new immune-metabolic viewpoint for age-related diseases. Nature Rev Endocrinol.

[41] Franceschi C, Campisi J (2014). Chronic Inflammation (Inflammaging) and Its Potential Contribution to Age-Associated Diseases. The Journals of Gerontology: Series A.

[42] Boule LA, Kovacs EJ (2017). Alcohol, aging, and innate immunity. J Leukoc Biol.

[43] Hammer AM, Morris NL, Earley ZM, Choudhry MA (2015). The First Line of Defense: The Effects of Alcohol on Post-Burn Intestinal Barrier, Immune Cells, and Microbiome. Alcohol Res.

[44] Kinirons MT, O'Mahony MS (2004). Drug metabolism and ageing. Br J Clin Pharmacol.

[45] Nyblom H, Berggren U, Balldin J, Olsson R (2004). High AST/ALT Ratio May Indicate Advanced Alcoholic Liver Disease Rather Than Heavy Drinking. Alcohol and Alcoholism.

[46] Kawaratani H, Tsujimoto T, Douhara A, Takaya H, Moriya K, Namisaki T (2013). The effect of inflammatory cytokines in alcoholic liver disease. Mediators Inflamm.

