# Supplementary material for: Hematological inflammatory biomarkers in patients with alcohol and cocaine use disorders
Source: Trends Psychiatry Psychother. 2025 Sep 18;47:e20230723. doi: 10.47626/2237-6089-2023-0723 (PMC12962387; doi:10.47626/2237-6089-2023-0723)
Supplement: Supplementary Material [file 2238-0019-trends-47-e20230723-suppl01.pdf]

http://doi.org/10.47626/2237-6089-2023-0723

Supplementary Table S1 Comparisons of NLR, MLR, and PLR biomarkers with clinical comorbidities, use of anti-inflammatory drugs, presence of HIV, HCV and/or syphilis in individuals with AUD

		Median (IQR)	n	p-value	Median (IQR)	n	p-value	Median (IQR)	n	p-value
		NLR			MLR			PLR		
HIV, HCV, and syphilis	Absence	1.97 (1.4-2.6)	390	0.191	0.35 (0.2-0.5)	390	0.442	105.33 (80.8-143.5)	385	0.696
	Presence	2.20 (1.5-2.9)			0.40 (0.2-0.5)			107.32 (75.9-171.6)		
Chronic diseases*	Absence	2.00 (1.4-2;5)	384	0.824	0.35 (0.2-0.5)	384	0.300	108.29 (81.1-143.9)	379	0.391
	Presence	1.94 (1.4-2.7)			0.37 (0.2-0.5)			98.54 (76.3-142.8)		
Anti-inflammatory medications†	Absence	1.99 (1.5-2.6)	390	0.519	0.36 (0.2-0.5)	390	0.497	107.11 (79.5-144.4)	385	0.839
	Presence	1.90 (1.2-2.8)			0.37 (0.2-0.5)			103.77 (79.9-136.5)		

AUD = alcohol use disorder; HCV = hepatitis C virus; HIV = human immunodeficiency virus; IQR = interquartile range; MLR = monocyte-lymphocyte ratio; NLR = neutrophil-lymphocyte ratio; PLR = platelet-lymphocyte ratio.
Data is presented as median (IQR).
* Chronic diseases: cardiovascular diseases, diabetes, stroke, cirrhosis, renal disease, respiratory problems, and autoimmune diseases.
† Anti-inflammatory medications: acetylsalicylic acid dipyrone, prednisone, ibuprofen, promethazine.

Supplementary Table S2 - Comparisons of NLR, MLR, and PLR biomarkers with clinical comorbidities, use of anti-inflammatory drugs, presence of HIV, HCV and/or syphilis in individuals with CUD

		Median (IQR)	n	p-value	Median (IQR)	n	p-value	Median (IQR)	n	p-value
		NLR			MLR			PLR		
HIV, HCV, and syphilis	Absence	1.71 (1.3-2.4)	583	0.356	0.30 (0.2-0.4)	583	0.539	115.28 (93.0-146.2)	579	0.513
	Presence	1.68 (1.3-2.1)			0.28 (0.2-0.4)			114.72 (87.6-146.5)		
Chronic diseases*	Absence	1.67 (1.3-2.2)	527	0.283	0.29 (0.2-0.4)	527	0.921	115.13 (93.2-146.4)	525	0.457
	Presence	1.77 (1.3-2.5)			0.30 (0.2-0.4)			112.94 (87.4-143.2)		
Anti-inflammatory medications†	Absence	1.69 (1.3-2.3)	583	0.455	0.29 (0.2-0.4)	583	0.243	115.28 (92.4-147.1)	579	0.140
	Presence	1.82 (1.3-2.9)			0.28 (0.2-0.3)			113.14 (85.1-125.4)		

CUD = cocaine use disorder; HCV = hepatitis C virus; HIV = human immunodeficiency virus; IQR = interquartile range; MLR = monocyte-lymphocyte ratio; NLR = neutrophil-lymphocyte ratio; PLR = platelet-lymphocyte ratio.
Data is presented as median (IQR).
* Chronic diseases: cardiovascular diseases, diabetes, stroke, cirrhosis, renal disease, respiratory problems, and autoimmune diseases.
† Anti-inflammatory medications: acetylsalicylic acid dipyrone, prednisone, ibuprofen, promethazine.
